# Myoelectric Control Systems for Upper Limb Wearable Robotic Exoskeletons and Exosuits—A Systematic Review

**DOI:** 10.3390/s22218134

**Published:** 2022-10-24

**Authors:** Jirui Fu, Renoa Choudhury, Saba M. Hosseini, Rylan Simpson, Joon-Hyuk Park

**Affiliations:** 1 Department of Mechanical and Aerospace Engineering, University of Central Florida, Orlando, FL 32816, USA; 2 Department of Electrical and Computer Engineering, University of Central Florida, Orlando, FL 32816, USA

**Keywords:** myoelectric control, EMG-based control system, upper limb wearable robot, upper limb exoskeleton, upper limb exosuit, biomechanical model, pattern recognition, machine learning, reinforcement learning

## Abstract

In recent years, myoelectric control systems have emerged for upper limb wearable robotic exoskeletons to provide movement assistance and/or to restore motor functions in people with motor disabilities and to augment human performance in able-bodied individuals. In myoelectric control, electromyographic (EMG) signals from muscles are utilized to implement control strategies in exoskeletons and exosuits, improving adaptability and human–robot interactions during various motion tasks. This paper reviews the state-of-the-art myoelectric control systems designed for upper-limb wearable robotic exoskeletons and exosuits, and highlights the key focus areas for future research directions. Here, different modalities of existing myoelectric control systems were described in detail, and their advantages and disadvantages were summarized. Furthermore, key design aspects (i.e., supported degrees of freedom, portability, and intended application scenario) and the type of experiments conducted to validate the efficacy of the proposed myoelectric controllers were also discussed. Finally, the challenges and limitations of current myoelectric control systems were analyzed, and future research directions were suggested.

## 1. Introduction

The demand for wearable robotic exoskeletons, more specifically upper limb rigid wearable robotic exoskeletons and soft wearable robotic exosuits, has substantially grown over the past few decades due to their promising applications across industry, and the medical and military sectors. The exoskeletons contain rigid links and joints attached to the user’s body, while exosuits use soft, flexible materials (such as fabric or soft polymer) to interface with the user’s body [1]. The intended application scenarios for upper limb exoskeletons and exosuits include: (i) power augmentation to enhance physical performance or the capabilities of able-bodied individuals during strenuous or repetitive physical tasks, (ii) assisting individuals with disabilities in performing activities of daily living (ADLs), and (iii) the rehabilitation of patients with neuromuscular disorders through therapeutic exercises [2].

**Figure 1:**
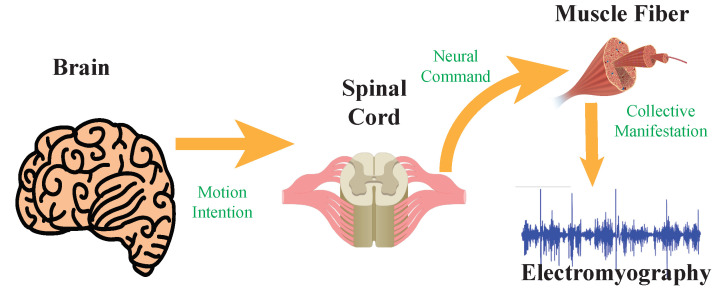
The generation of electromyography signal corresponding to the muscle contraction.

The myoelectric control systems of upper limb exoskeletons are based on surface electromyography (EMG) signals, which are the electric potentials directly measured from skeletal muscles and that are generated from motor unit activation [3]. The generation of EMG signals is controlled by the human brain through motion intention, and is regulated by motor neurons in the spinal cord (Figure 1), which offers a means for detecting the human motion intention before initiating a motion [4]. Compared to other control systems, the critical advantage of myoelectric control is its timely detection of the user’s motion intention leveraging electromechanical delay; the onset of motion intention can be detected about 50–100 ms earlier than the physical motion [5,6], giving time to control the upper limb wearable robotic exoskeletons to allow for more adaptive and intelligent human–robot interactions [7].

The early stages of myoelectric control modalities for upper limb exoskeletons were based on on-off/finite state control and proportional control [8,9]. Although they are fast, reliable, and applicable for real-time operation, the number of movements generated by these controllers is limited [10]. Therefore, they have been mostly utilized in supporting simple upper limb functions such as elbow flexion/extension or power grip. Recent advancements in machine learning (ML) and deep learning algorithms have made it possible to decipher more complex movements over multiple DOFs (such as shoulder motions) by extracting useful features from s-EMG signals [11]. Although they have shown promising results in supporting complex, multi-DOF upper limb motions, their development is still in the early phase.

Given the growing demand for exoskeletons, the number of publications on myoelectric control systems for upper limb exoskeletons and exosuits has rapidly increased over the past decade. It is therefore imperative to understand the latest trends and developments of myoelectric control systems for upper limb exoskeletons and exosuits, and to analyze their advantages and limitations in order to pinpoint the future directions for advancing this field. However, to the best of our knowledge, there has not been any comprehensive review that specifically focuses on the myoelectric control systems of upper limb exoskeletons and exosuits. Therefore, this review provides a comprehensive overview that will serve as a guidance for researchers and developers working in this field to address the challenges and limitations of existing myoelectric control systems, which in turn will enhance the utility and practicality of this technology and boost its real-world transition.

This review includes articles published in IEEE Xplore, Web of Science, and PubMed. Because the search result indicates that 194 out of 228 research articles were published between 2011 and 2021, this review will only focus on the research articles published during this duration. This review particularly focuses on the state-of-the-art myoelectric control systems designed for upper limb exoskeletons successfully implemented on a physical platform, i.e., exoskeleton/exosuit prototypes, and validated experimentally. The rest of the paper is organized as follows: Section 2 describes the methods used in conducting the literature review (e.g., eligibility criteria, search query, and screening). Section 3 summarizes the results of crucial design aspects, different myoelectric control modalities, and experimental validation methods found in the literature. Section 4 discusses existing challenges and outlines future research directions, and Section 5 provides the conclusion.

## 2. Methods

The method used to search and to identify key literature pertaining to myoelectric control systems on upper limb exoskeletons are described in this section. This process was guided by the PRISMA method [12] for conducting a systematic review of the literature, based on specific search criteria and objectives.

### 2.1. Eligibility Criteria

The eligibility criteria of this survey include articles: (i) written in English, (ii) published between 2011 and 2021, (iii) using s-EMG signals as inputs to control the upper limb wearable robotic exoskeleton, and (iv) implementing the controller on a physical model and conducting an experimental validation on human subjects. The time frame selected for this survey (criteria (ii)) was to capture the most recent advancements in myoelectric control systems for upper limb wearable robotic exoskeletons within the past 11 years. In addition, criterion (iv) was set to preclude studies in which myoelectric controllers were realized only in computational models or through simulation, since they cannot capture some uncertainties and nonlinearities present in the human–robot interface in the real world [13,14].

### 2.2. Keywords and Search Query

The research articles reviewed in this paper were collected from three databases: IEEE Xplore, Web of Science, and PubMed, which are the database for the world’s highest quality technical literature in engineering and technology (IEEE Xplore), the world’s oldest, most widely used, and authoritative database of research publications and citations (Web of Science), and the world’s largest free MEDLINE database for life sciences and biomedical literature (PubMed).

All three databases accepted similar search queries and keywords with small variations (Table 1). The keywords used were: (i) Body segments: “Upper Limb”, “Upper Body”, “Arm”, “Shoulder”, “Elbow”, and “Forearm”; (ii) Types of Exoskeletons: “exoskeleton”, and “exosuit”; (iii) Electromyography signal: “Electromyography”, “Myoelectric”, “Myoelectric control”, “Surface Electromyography”, “EMG”, and “EMG”.

**Figure 2:**
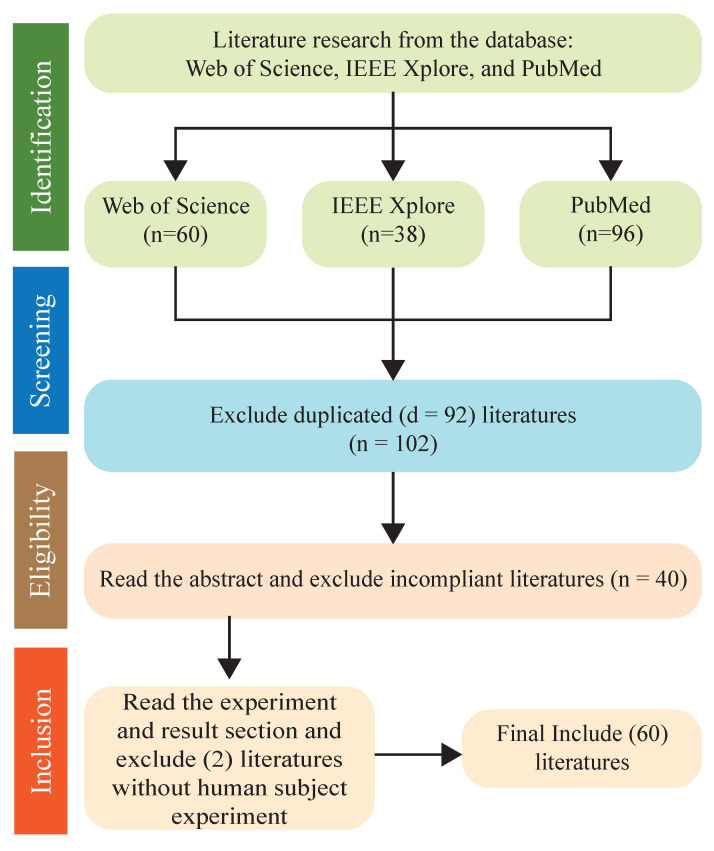
Process of searching, screening, filtering, and the selection of research articles to be included.

The search query resulted in 180 articles, among which 90 were counted twice, as they appeared from more than one database. In addition, based on criteria (iv), i.e., the implementation and validation of the proposed myoelectric control system on a physical model, 30 articles were excluded. Finally, 60 articles were selected and reviewed in this survey. Figure 2 illustrates the https://www.overleaf.com/project, (accessed on 12 September 2022) process of searching, screening, filtering, and the selection of articles to be included [12].

## 3. Results

This result section contains the description and analysis of the included research articles. A narrative style is adopted to present our results. The results are presented based on four aspects: (i) a general description of information sources, (ii) different types of myoelectric control systems, (iii) key design characteristics of the device (i.e., DOF, portability, and intended application scenario), and (iv) types of experiments conducted to evaluate the efficacy of the proposed controller.

### 3.1. Overview of Information Sources

A summary of the relevant characteristics of the 60 articles included in this literature review is organized in Table 2, which includes: bibliographic citation and information of authors of the article (Ref and Author), publication year of the article (Year), upper limb segments assisted by the exoskeleton (Body Segment), types of myoelectric control method (Control Method), portability of the device (Device Portability), supported degree of freedom (DoF), intended application scenario (Application of Device), and number and health condition of subjects in experimental validation (Experimental Subject).

**Figure 3:**
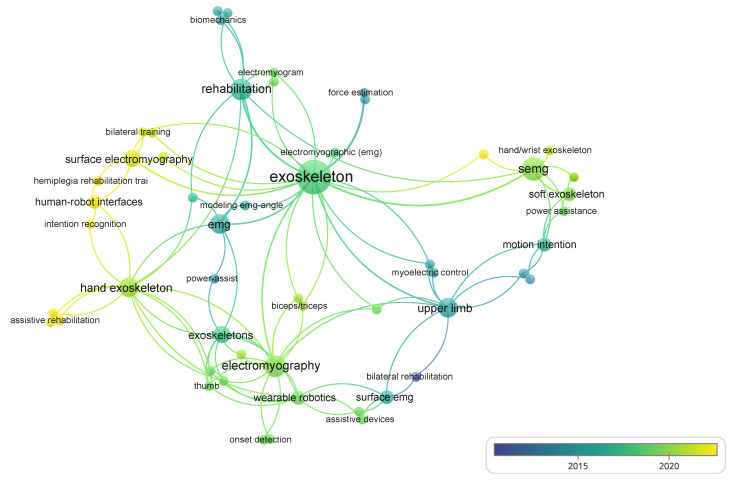
The chronicle evolution of the topics related to the myoelectric control system of upper limb exoskeleton and interrelationship between each keywords. The map is generated with VOSViewer [73].

In addition, we conducted a bibliometric analysis of the selected articles and plot (Figure 3) to present the network of relationships between the keywords of the myoelectric control system and upper limb wearable robotic exoskeletons associated with the documents examined. The bibliometric analysis reveals the evolution of the topics related to the myoelectric control system of upper limb exoskeletons over time, and their connections with other key terms.

### 3.2. Taxonomy of the Myoelectric Control System for Upper Limb Robotic Wearable Exoskeletons

**Figure 4:**
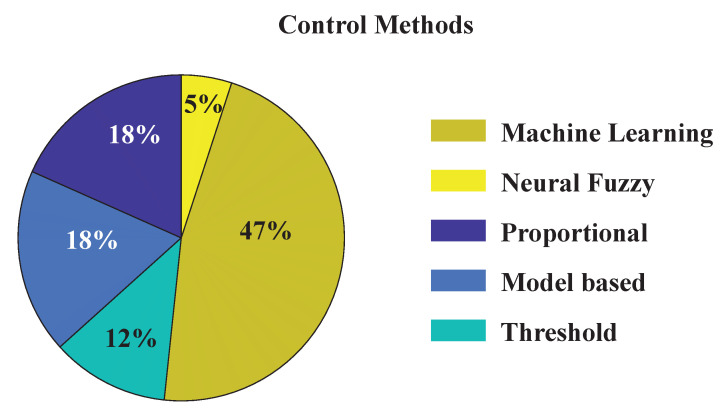
The percentage distribution of various types of myoelectric control systems among the included research literature, which includes biomechanic model-based, machine learning-based, proportional, threshold-based, and neural-fuzzy-based myoelectric control systems.

The myoelectric control system is designed to detect the movement intention of the user by deciphering the EMG signals generated in skeletal muscles. Among the reviewed literature, different modalities of myoelectric control systems for upper limb exoskeletons were identified (as shown in Figure 4), which include: (i) Threshold-based control (12%, seven articles), (ii) Proportional Control (18%, ten articles), (iii) biomechanical model-based control (18%, ten articles), (iv) machine learning and deep learning-based control (47%, twenty-eight articles), and (v) neural-fuzzy control (5%, three articles). The ML and deep learning-based myoelectric control systems were further sub-categorized into (a) pattern recognition or classification-based control, (b) simultaneous or regression-based control, or (c) reinforcement learning-based control.

#### 3.2.1. Threshold-Based Myoelectric Control

**Figure 5:**
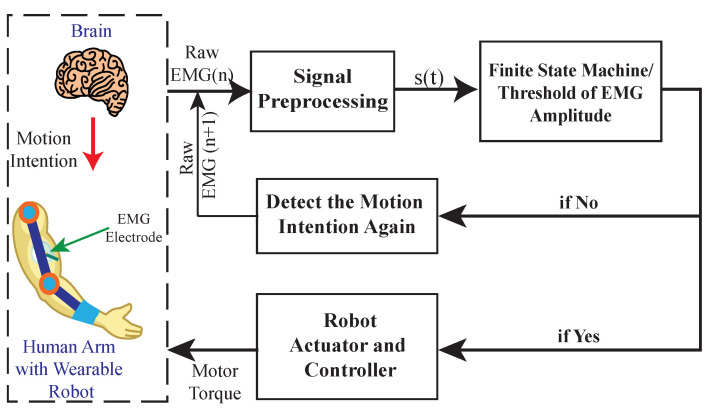
The conceptual block diagram for threshold-based (On-off/Finite State Machine) myoelectric control system.

Threshold-based myoelectric control is a method that regulates specific control rules by comparing the amplitude [31] or statistical features [15] of EMG signals with certain thresholds to assist the user (as shown in Figure 5). The studies at [38,60] implemented threshold-based myoelectric control to assist the user in the flexion and extension of the forearm with different loads in hand. The proposed controller utilized a Double Threshold strategy based on EMG signals corresponding to Bicep Brachii (BB) and Triceps Brachii (TB). When the user tried to flex his/her arm with a load, the EMG signal corresponding to BB exceeded its threshold value (i.e., the amplitude of BB signal at the no-load condition) which activated the exoskeleton to support the user in the intended motion. Similarly, during forearm extension motion under load, the EMG signal corresponding to TB was greater than its threshold (i.e., the amplitude of TB signal at the no-load condition), which triggered the exoskeleton to assist the user during the performance of extension. Experiments in [38,60] validated the proposed control strategy under various load conditions with the same threshold values. It was demonstrated that the exoskeleton could successfully assist the user in supporting the motion by reducing his/her muscle effort, showing the adaptability of this strategy under different load conditions.

To improve the accuracy of threshold-based control, [30,45] utilized sensor fusion methods are utilized to determine thresholds to modulate assistance. In [45], signals from both EMG and force-sensing resistors (FSRs) were used to detect the user’s motion intention. By measuring the contact force between the user and the robot, FSRs were utilized to detect the user’s motion intention. When both EMG and FSR signals exceeded their thresholds, the assistance of a wearable robotic exoskeleton was engaged. In [45], the end-effector position (measured by IMU sensors), joint torques (estimated from force/torque sensors), and EMG signals were used in the sensor fusion method. The proposed control strategy in [30] was intended to assist patients with upper limb impairment in pointing his/her arm to the desired position; therefore, a fusion of end-effector position with joint torques and EMG signals enabled the user to track his/her desired trajectory more accurately. Same as [45], the conditions for controlling assistance in [30] were the end-effector position not being at the target position, and both joint torques and amplitudes of EMG signals being greater than their threshold values. Overall, threshold-based myoelectric control systems have the lowest computational cost. However, this control strategy can only support a limited number of movements at a time and is sequential in nature, unlike the continuous natural movement of the human upper limb.

#### 3.2.2. Proportional Myoelectric Control

**Figure 6:**
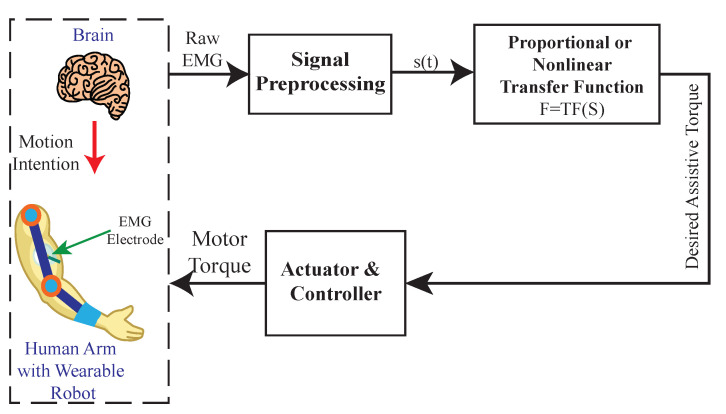
The conceptual block diagram for proportional/directed myoelectric control system.

Proportional myoelectric control utilizes a transfer function to continuously map the processed EMG signals to the dynamics of upper limbs (e.g., force, velocity, position, etc.), then uses it as a control input to assist the user in performing a specific movement (as shown in Figure 6). Whether the EMG signal is directly mapped to the desired joint angle or torque, this control scheme is divided into direct and indirect proportional control.

Direct proportional control scales the processed EMG signal to the muscle effort because the amplitude of EMG signals infer the amount of muscle contraction [56,57] and estimate the generated joint torque [58]. Based on this notion, one can develop a direct myoelectric control system that maps the amplitude of EMG signals with a scaling factor to estimate the user’s effort, which can then be used as a control input. The implementation of a direct proportional myoelectric control system for upper limb wearable robotic exoskeletons was first introduced by Lenzi et al. [9]. This work used a proportional gain as a scaling factor to estimate the user’s effort using EMG signals. The estimated user’s effort was then used as input to control the assistance of a 1-DOF elbow exoskeleton. The proportional gain was tuned manually with the control goal of the gravity compensation of the arm and the carried load, while performing repetitive arm motions. The experiment showed an average reduction of 31% in the user’s muscular effort, with acceptable accuracy in maintaining the desired trajectory. In addition to [9,74], a similar approach has been implemented by other studies [19,43,44,48] on hand and wrist exoskeletons, and similar performances of controllers were found. However, in these studies, an additional low-level controller was required to regulate the actuator. In [34], the amplitude of EMG signals was scaled to the duty cycle of a PWM controller, and the myoelectric control system was directly able to regulate the actuator. Compared to [9,19,43,44,48], the subsequent approach required less computational power, improving the exoskeleton’s portability and useability.

Linear transfer functions were used to map the EMG signals in [9,19,43,44,48], but [62] used a nonlinear transfer function to represent the relationship between joint torque and EMG signal [75]. This nonlinear function was derived using experimental data; therefore, it is expected to provide more accurate joint torque estimation. Apart from using a nonlinear transfer function for joint torque estimation, the myoelectric control system in [62] also implemented a neural network model to estimate the direction of joint torque using the EMG signal. Other studies implementing indirect proportional control in the literature [39,61] utilized an admittance control system to map the EMG signal to the desired joint kinematics (i.e., joint angle or angular velocity). Compared to direct proportional myoelectric control systems, indirect myoelectric control systems have been shown to improve the trajectory tracking performance of the upper limb wearable robotic exoskeletons. Overall, proportional myoelectric control is easier to implement and shows robust performance in assisting the user in performing simple motion tasks such as elbow flexion/extension. The limitation is that it is not suitable for supporting complex, sophisticated multi-joint motion tasks in patients with upper limb impairment.

#### 3.2.3. Biomechanical Model-Based Myoelectric Control

**Figure 7:**
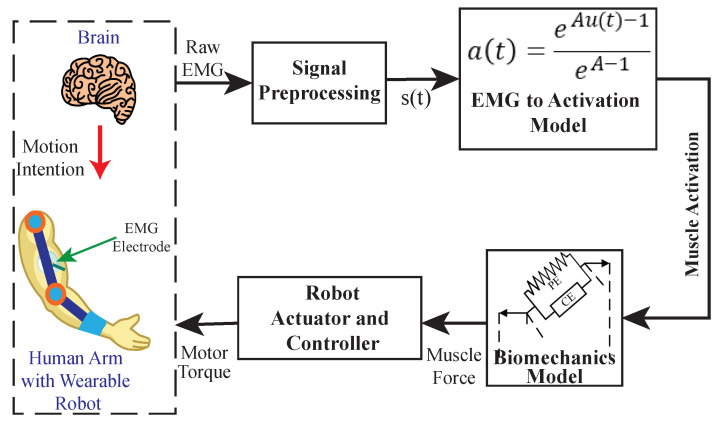
The conceptual block diagram for bio-mechanics model-based myoelectric control system.

A biomechanical model-based control system employs a musculoskeletal model to map the EMG signals to the desired control inputs, where the human musculoskeletal model is represented by a mechanical multi-body system actuated by muscle segments (as shown in Figure 7). Hill’s Muscle Model was the only biomechanical model identified in the reviewed literature that was utilized in myoelectric control of upper limb exoskeletons.

The survey results indicated that only a small portion of the reviewed articles (about 18%, 10 articles) used biomechanical model-based myoelectric control systems, and most were intended for power augmentation. The first implementation of Hill’s Muscle Model in the myoelectric control system was presented by [76] to control a single DOF upper limb exoskeleton to assist the user in elbow flexion/extension. The control system used Hill’s Muscle Model to estimate the moment generated by the Bicep Brachii and Triceps Brachii medial-head muscles as a primary input to the controller. The exoskeleton’s kinematic and dynamic effects were examined experimentally on the assisted arm segments [77]. The results showed a reduction of around 86% in muscular activity and joint torque using the assistive system.

The authors in [59] also implemented a similar approach to develop an adaptive biomechanical model-based myoelectric control system for a one-DOF upper limb exosuit. The purpose of the proposed myoelectric controller was to estimate the joint torque without using the inverse dynamic model. Instead, Hill’s Muscle Model was implemented to map the EMG signal to the joint torque, and then the estimated joint torque was used as control inputs to a closed-loop admittance controller to detect motion intention and to compensate for the estimated joint torque. Their experimental results indicated that the proposed myoelectric control system had an average coefficient of determination ($r^{2}$) 0.87±0.04 (mean ± SD) across different human subjects, which demonstrated the robustness of the proposed system. Furthermore, for elbow motion assistance under various loads, the average $r^{2}$ was 0.92±0.03, which demonstrated the good adaptability of the system.

One limitation of Hill’s Muscle Model is that there are situations when it cannot yield an accurate detection of the user’s motion intention [78]. For example, there are cases where muscle activations occur without necessarily involving joint movement (e.g., isometric contraction) [79]. Therefore, a myoelectric control system using Hill’s Muscle Model could misinterpret the user’s motion intention, resulting in incorrect control commands. To address this limitation, Lotti et al. [59] introduced an additional encoder installed at the elbow joint to detect the kinematic velocity of elbow flexion/extension, then used them as another input signal for the controller. On the other hand, Liu et al. [63] introduced another biomechanical model-based myoelectric control system with the capability of movement onset detection, but only with the use of the EMG signal as input. The proposed myoelectric control system in [63] combined Hill’s Muscle Model with a support vector machine (SVM) to control an upper limb exoskeleton for load-lifting assistance. The Hill’s Muscle Model was used to map the EMG signal to the joint torque and it was used as the control command, similar to [59]. In addition, an SVM-based binary classifier was also incorporated for detecting the movement onset, and it activated the actuator once the movement onset was detected. Compared to [59], the integration of Hill’s Muscle Model and an SVM-based classifier eliminates the delay in the control system while ensuring its accuracy. However, some limitations of the approaches in [59,63] include repetitive calibrations for different subjects and/or tasks, a long setup time, a high computational cost, and a lack of continuous control [80], and it requires many muscle segments for complex joints (e.g., shoulder joint) [81].

To reduce the setup time and to use fewer numbers of muscle segments for the shoulder joint muscle, ref. [23,29] used an optimization algorithm to tune the parameters in Hill’s Muscle Model. Different from the conventional calibration approach that used the human’s anatomical model [82], this work measured the arm’s gravitational torque and derived the expression of muscle torque from Hill’s Muscle Model, and then it used a Genetic Algorithm (GA) to optimize the parameters of the muscle model by minimizing the difference between the gravitational torque and muscle torque. Another study in [47] compared the performances of the GA and Linear Optimization Algorithms in tuning the parameters of Hill’s Muscle Model. The results indicated that Hill’s Muscle Model, tuned using a GA, showed a higher accuracy in estimating the joint torque ($R^{2}=0.9$ for GA vs. $R^{2}=0.8$ for Linear Optimization). Furthermore, compared to [59], which required one extra session to calibrate the muscle model, the GA optimizer in [23,29] only required two minutes to find the optimal parameters of the muscle model. Even though the work in [23,29] significantly reduced the calibration time compared to [59], the results still showed an average reduction of 67% in the user’s muscular effort for the load lifting task, and an average increase of 8% in the $R^{2}$ score after the GA optimization.

The research articles mentioned above use Hill’s Muscle Model to estimate the joint torque; however, it can also be used in the myoelectric control system for other purposes. For example, ref. [37] developed a human-in-the-loop adaptive impedance control system for an elbow exoskeleton. The exoskeleton in [37] is designed for upper limb rehabilitation; therefore, both the predefined trajectory and the EMG signals were used as the control command. To adapt the control gains to the human muscle’s impedance, this work used Hill’s Muscle Model to calculate the muscle stiffness in real time, which is proportional to the joint torque from the measured EMG signals. The benefit of such adaptive impedance control systems in the upper limb rehabilitation task is the accuracy of its output trajectory with respect to the desired trajectory (which is almost close to 100%).

Furthermore, ref. [35] used the EMG signal and Hill’s Muscle Model as a bio-feedback of the myoelectric control system. Hill’s Muscle Model was still used as a joint torque estimator in this work, similar to [29,47,59]. However, it used a predefined trajectory as a control command, and the goal was to minimize the biofeedback measured from the EMG signal. The minimization of biofeedback is targeted to reduce the error between the predefined trajectory and the actual trajectory.

As validated in many studies [24,29,35,37,47,59,63,72] the model-based myoelectric control systems are effective and versatile in controlling upper limb wearable robotic exoskeletons using EMG signals as the control input. Nonetheless, some limitations in the model-based myoelectric controls remain, such as a high computational cost, requiring repeated calibrations, and not being robust to unmodeled and/or external disturbances.

#### 3.2.4. Machine Learning-Based Myoelectric Control

**Figure 8:**
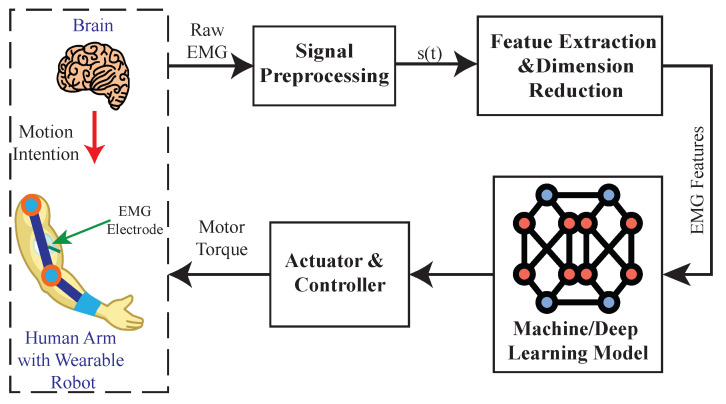
The conceptual block diagram for deep learning/machine learning model-based myoelectric control system.

The machine learning approach has been employed in upper limb exoskeletons to predict the movement pattern from EMG signals using various modalities. Such modalities of the myoelectric control system use the statistical features of the preprocessed EMG signals, such as root mean square (RMS), wave length (WL), and mean absolute value (MAV) [83] as inputs to different machine learning models, detecting the type of movement via classification model [21,49], measuring the arm’s joint angle or joint torque via regression model [27,32], and minimizing muscle effort during assistance with reinforcement learning [40] (as shown in Figure 8).

1 Pattern recognition or classification-based myoelectric control
  In the myoelectric control system, the classification model can detect the type of movement corresponding to the input EMG signal. For example, ref. [49] implemented the SVM algorithm to predict whether the user’s finger is in motion or not via the root mean square features of the EMG signal; the authors in [21] implemented a type of decision-tree algorithm named MCLPBoost to predict whether the elbow and wrist are performing downward or upward movements. In the abovementioned studies, only a single type of classification algorithm was used, and a comparison between different algorithms was not conducted. To compare the performance of different classification algorithms implemented on a hand exoskeleton, the authors in [84] compared the accuracy of four classification algorithms, i.e., SVM, artificial neural network (ANN), linear discriminant analysis (LDA), and K-nearest neighbors (KNN), to predict the EMG features to five types of hand movements, and then used the classification result as an input of the assistance controller. The results showed that SVM had the best accuracy among those four in predicting the type of hand movements. To improve the performance of the classification model, studies such as [8,50] compared two types of classifiers with same algorithm but different input signals. The type 1 classifier used a single channel EMG signal but converted it to 14 different statistical features; the type 2 classifier used EMG signal from five channels and only converted them to a single statistical feature. Both type 1 and 2 classifiers were designed to detect if the arm was in motion or not. The results indicated that the type 1 classifier had higher accuracy, but the type 2 classifier had less latency. Refs. [8,50] indicated that extracting the EMG signal to multiple features can improve the accuracy of a classification model. Another two studies [28,52] used a sensor fusion method that used both EMG and electroencephalography (EEG) signals to improve the accuracy of the classification model, and [46] implemented a threshold method in which the signal amplitudes must be greater than a predefined threshold in order to be input to the classification model. Such a threshold method can prevent misclassification and can improve the accuracy of myoelectric control systems.
2 Regression-based Myoelectric Control
  The regression model represents the relationship between input and output data as a function that is trained with a pre-collected dataset. Compared to the classification model, which can only detect discrete motions, the regression model can output continuous variables with the statistical feature of the EMG signal as input, such as joint angle and joint torque. The regression models can be constructed using different approaches, such as linear regression, ANN [27], and Kalman Filters [57]. Because of the nonlinearity of the EMG signal, the articles included in this review only used ANN or the Kalman Filter as the regression model.
  Among the included articles, ref. [27] implemented a machine learning-based myoelectric control system to control an elbow exoskeleton by training a back propagation neural network (BPNN) to estimate joint angle from the statistical feature of the user’s EMG signal, and showed that the regression model could accurately estimate the user’s joint angle. Another application of such machine learning-based myoelectric control systems was bilateral hand training with a wearable hand exoskeleton, which used the statistical feature of an EMG signal from an unimpaired hand to estimate the magnitude of the assistive force, to train the impaired hand. In this review, research articles such as [18,20,22,32,46,56,69], used neural network-based regression models as a myoelectric control system to control a hand exoskeleton for training different parts of the impaired hand. On the other hand, ref. [57] used a Kalman filter-based regression model to compute the joint torque based on the EMG signal. Compared to the neural network model, the Kalman filter model does not need much time, nor extensive datasets to train the model. Moreover, tuning the Kalman filter model for different users only requires the measurements of a few sets of joint torque under different positions. Although the regression model in [57] reduced the effort in training the model, its accuracy and efficacy in assisting the upper limb were similar to that of the neural network-based regression model. Based on the adaptive and robust regression model developed in [57], ref. [52] integrated a neural network and a Kalman filter-based regression model in which the neural network took multiple variables as inputs, which includes processed EMG signals, the joint torque estimated by the Kalman filter, and the joint angle and joint angular velocity measured by the IMU sensor, to control the motion of the upper limb exoskeleton. The myoelectric control system proposed in [52] achieved a better accuracy and efficacy compared to the previous regression models explained above.
3 Reinforcement Learning-based Myoelectric Control
  In addition to the classification and regression model, the reinforcement learning model was also used in the myoelectric control system in the reviewed articles. Unlike the classification and regression models, the reinforcement learning model is trained with a reinforcement learning algorithm that uses a smart agent to learn the optimal policy while interacting with an environment. During the process of reinforcement learning, the smart agent exerts an action on the environment, based on its observation of the environment (state) as feedback, and then the environment returns a score to evaluate the agent’s action (reward). Based on the reward returned from the environment, the agent optimizes its policy toward the direction of greater reward [85].
  In the field of wearable robotics, reinforcement learning has been used in prosthetic control (e.g., [86]), lower limb exoskeleton control (e.g., [87]), and joint torque estimation (e.g., [88]). Compared to other types of control methods used in EMGs, the reinforcement learning algorithm reflects the interaction between humans and the environment. In addition, reinforcement learning can provide an optimal control policy without the knowledge of the environment, which is ideal for use in complex and uncertain environments. Hamaya et al. [40] used a reinforcement learning algorithm called Probabilistic Inference for Learning Control (PILCO) to control an elbow exoskeleton. The state vector consisted of the kinematics of the elbow joint and EMG signals, and the reward was related to the difference between the desired and actual trajectory. The PILCO algorithm implemented the Gaussian process to learn the probabilistic dynamic model of the human–exoskeleton interface through the states collected during human–exoskeleton interaction, then evaluated the control policy using the learned probabilistic dynamic model, and finally optimized the control policy through the policy gradient method [89]. This method offered a faster training time as compared to other machine learning myoelectric control systems.
  As mentioned above, the ML-based myoelectric control schemes show promising results in increasing the accuracy and reliability of myoelectric control of an upper limb wearable robotic exoskeleton. However, some limitations exist in the ML-based myoelectric controls, such as a limited implementation in multi-DOF upper limb wearable robotic exoskeletons [9], poor performance in online training of the machine learning model [90], and high computational cost [53].

#### 3.2.5. Neural-Fuzzy Myoelectric Control

**Figure 9:**
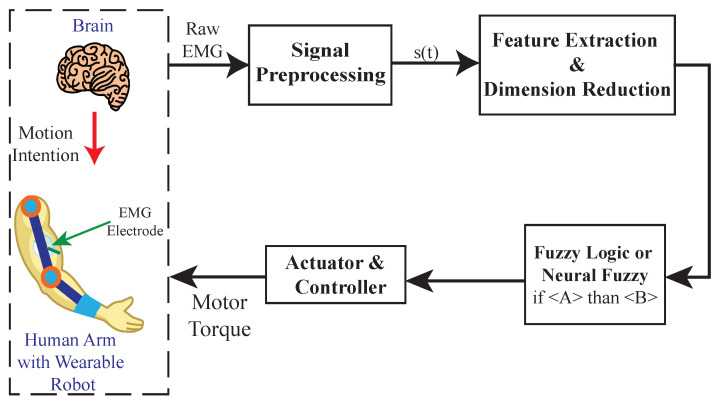
The conceptual block diagram for neural-fuzzy-/fuzzy-logic-based myoelectric control system.

The neural-fuzzy-based myoelectric control system is another scheme for controlling an upper limb exoskeleton-type exoskeleton. According to the theory of modern artificial intelligence, neural fuzzy is defined as the combination of a neural network and fuzzy logic [91]. The fuzzy logic outputs the control command while the neural network tunes the fuzzy logic iteratively, which allows for training of the fuzzy logic during use (i.e., fuzzy modifier) (as shown in Figure 9).

José et al. [92,93] first introduced the neuro-fuzzy myoelectric control system, where the fuzzy logic consisted of “IF <premise set> and THEN <consequent set>” statements, and the fuzzy modifier was a fully connected neural network. The premise set in this fuzzy logic consisted of the motion status of EMG signals from eight muscles, and the consequent set included the desired motor torque and the gain of controllers. The fuzzy logic needed to be tuned by the neural network using the EMG signals. The tuning process was similar to training a neural network that uses backpropagation and activation functions. Since the fuzzy logic is trained each time before making a control command, it makes it adaptive to various environments. This type of neuro-fuzzy myoelectric control system has been adopted by [16,92], and the results have shown that it reduces the user’s muscle activation during various activities of daily life. However, the notable downside of this approach is that the fuzzy logic becomes complicated as the number of DOFs of the exoskeleton increases. When a exoskeleton has multiple DOFs, the number of fuzzy logics will increase exponentially, consequently increasing the difficulty in implementing and training fuzzy logics.

To improve the compatibility of the neuro-fuzzy myoelectric control system for higher DOF exoskeletons, a different type of fuzzy logic was implemented on a 7-DOF upper-limb exoskeleton [17], which represented fuzzy logic as a matrix product of the EMG signal from 16 channels and the weights of each neuron in the fuzzy modifier. Similar to other studies, the fuzzy modifier was a fully connected neural network tuned every time before running. The experiment of this study showed that under the assistance of a wearable robotic exoskeleton, the muscular efforts of each muscle segment were reduced.

### 3.3. Key Design Characteristics

This section presents the literature research for the design aspects of upper limb exoskeletons that are equipped with the myoelectric control system, which include degrees of freedom, portability, and the application of upper limb exoskeletons.

#### 3.3.1. Degrees of Freedom

**Figure 10:**
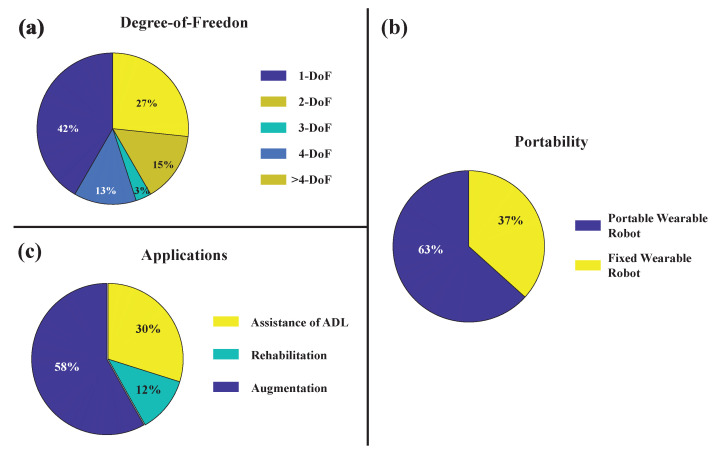
(**a**) The percentage of the degrees of freedom for the upper limb exoskeletons in the included research literature; (**b**) The percentage of the applications of the upper limb exoskeletons in the included research literature; (**c**) The percentage of portability of the upper limb exoskeletons in the included research literature.

As shown in Figure 10a, 13% (seven articles) of the studied articles have worked on controlling two DoF systems. Additionally, 3%, 15%, and 27% (1, 9, and 16 articles, respectively) of the explored studies pertain to the three, four, and greater than four DoF robots. Considering the single DoF exoskeletons (42% of the reviewed papers and 25 articles), many have exclusively studied the flexion and extension of the elbow joint (Table 2). During testing protocols, they may require the subjects to make different selected angles wearing the arm device while handling varying load weights [9,35]. The biceps muscle responsible for elbow flexion and extension motion would then be used as the control signal. In some studies, while flexion actuation is achieved through the device, gravity is responsible for extension actuation [23,25]. Many papers have focused on the actions of the fingers or that of the wrist (Table 2), within their robotic hand device. A hand exoskeleton can be activated simply through two independent DoFs, one for the thumb and one for the four fingers’ flexion/extension [65]. Alternatively, for grasping movement, the thumb can passively be fixed to its position [20,32]. Soft robotic gloves are also a popular field of research, where the production of sufficient forces to grasp objects through the robotic glove is studied [19].

#### 3.3.2. Portability of Upper Limb Robotic Wearable Exoskeletons

Among the reviewed literature, 63% (37 articles) of studies developed upper limb exoskeletons with portable structures, while the remaining ones presented fixed upper limb robots (as shown in Figure 10c). Portability is an important design requirement for exoskeletons that have intended applications in the performance augmentation of able-bodied individuals or for the assistance of older adults and patients with neuromusculoskeletal disorders in activities of daily life. For rehabilitative use, exoskeletons need not be essentially portable if they are being utilized in clinical environments or therapeutic institutes. However, the portability of rehabilitative robots is desired for home-based rehabilitation environments since they can provide ease of access and more flexibility to the user, compared to stationary robotic systems.

The majority of studies that presented portable upper limb robots were based on hand exoskeletons [15,18,19,20,22,32,41,42,43,44,46,48,49,54,55,56,65,69,72]. There were also a number of studies that developed portable exoskeletons/exosuits to support elbow movement [17,23,27,31,38,39,45,52,57,58,59,60,61,64], or both hand and elbow motions [24,28,51]. Only one portable exoskeleton was found in the literature that provided power assistance for the flexion/extension movement of both elbow and shoulder joints [71]. Most of the portable upper limb robots mentioned above had self-contained architecture. Only a few powered exoskeletons assisting elbow movement [24] or elbow–shoulder movements [71] required the user to wear backpack support while operating the device.

When an upper limb exoskeleton is designed to support movements of more than one joint, the number of required actuators and the weight of the robot both increase. Therefore, most of the exoskeletons supporting motions of two or multiple joints, such as shoulder–elbow [29,47], elbow–wrist [8,26], or shoulder–elbow–wrist [16,17], found in the literature, were not portable. Apart from that, some studies also mentioned fixed exoskeletons supporting a single joint motion, which were mainly elbow exoskeletons [9,17,21,25,30,33,34,37,40,47,53,62,63] (except one shoulder exoskeleton in [68] and one hand exoskeleton in [36]), which were not portable because of their high weight-to-power ratio.

#### 3.3.3. Application of Upper Limb Robotic Wearable Exoskeletons

The potential application scenarios identified in the reviewed literature can be broadly categorized as rehabilitative use (58%, 34 articles), assistive use (30%, 18 articles), and human augmentation (12%, eight articles) (as shown in Figure 10b). Rehabilitative upper limb exoskeletons can aid patients with motor disabilities in performing therapeutic exercises, in order to restore/improve the motor functions in their impaired limbs. A total of 58% (34 articles) of the exoskeletons in the reviewed papers were intended for rehabilitation scenarios, assisting the patient in performing different single or multiple joint motions (some examples include [15,16,21,24,29,68]). The benefits of wearable robot-assisted rehabilitation compared to traditional physical therapy include the provision of repetitive and intensive training to the patient, lessening the physical burden of the therapist, and offering an effective means of objectively quantifying the patient’s progress before and after training [1].

About 30% (18 articles) of the reviewed literature developed assistive upper limb wearable robotic exoskeletons (e.g., [22,26,36,41]), which were intended to support people with neuro-muscular conditions (such as stroke, spinal cord injury, muscle weakness, etc.) as well as older adults in performing the activities of daily living (ADLs). Such assistive robots may enable users to regain some of their functional independence, facilitate their participation in daily activities and overall, enhance the quality of their lives. Furthermore, some wearable robotic exoskeletons (e.g., [17,35,63]) have been designed for rehabilitation and assistance scenarios to be utilized in physical therapy in clinical environments, or to provide assistance during ADLs.

Only 12% (eight articles) of the reviewed exoskeletons were developed for potential application in human performance augmentation [31,39,50,71], which can enhance the strength, endurance, or physical capabilities of healthy individuals during repetitive and/or strenuous tasks in factories, warehouses, or military bases or excursions. Using these devices can reduce the risk of developing musculoskeletal disorders in industry workers and military personnel, as well as reducing their metabolic costs during lifting or carrying of heavy loads.

### 3.4. Human-Subject Evaluation of the Myoelectric Control System

Among the reviewed literature, nine studies tested their prototypes on subjects with neuro-muscular impairment (either stroke or SCI patients) [15,24,32,36,41,54,64,65], while the remaining ones performed experimental validations on healthy subjects. The validation tests found in the reviewed studies can be broadly grouped into the following categories: (i) kinematic evaluation, (ii) user’s effort evaluation, (iii) ML model performance evaluation, and (iv) clinical assessment.

The controller performance was tested in many studies using kinematic evaluation, where the most commonly used outcome measures identified were trajectory tracking performance (i.e., joint angle tracking) and velocity [9,16,17,21,22,24,57,61,62,65,67]. Several studies tested the proposed controller’s efficacy by evaluating the user’s effort in performing a specific movement or task [9,17,23,57,60,63]. To do so, EMG signals from relevant muscles were measured during static/dynamic tasks to estimate muscle activation and EMG-derived joint torque. Then, comparisons were made among different scenarios (such as not wearing the exoskeleton, wearing it without assistance, and wearing it with assistance under the same or different loads) to quantify the reduction in muscle effort. In addition, a few studies utilized isometric contraction tests to evaluate the effect of the developed prototype on muscle fatigue [23,64]. Classification accuracy and confusion matrix outcomes commonly evaluated the ML-based myoelectric control systems’ performances and computation times (or latencies) [18,63,64,67,68,69]. Only one study evaluated the learning time for a model-based reinforcement learning framework, and found that the proposed method could learn the proper assistive strategy after only 60 seconds of user–robot interaction [40].

Only one study [15] performed a clinical assessment on eight chronic stroke subjects using the Fugl-Meyer Assessment (FMA) and the Action Research Arm Test (ARAT) to evaluate the improvement in hand and upper arm functions, respectively, after 20 sessions of hand exoskeleton-assisted training. In addition, the Sollerman Hand Function Test was used in another study [41] to evaluate the hand performance of two SCI patients with and without wearing the active hand exoskeleton.

## 4. Discussion

In this review, myoelectric control systems designed, implemented, and validated on upper limb exoskeletons, especially the rigid-link exoskeleton and exosuit, were classified into threshold-based, proportional, biomechanical model-based, machine learning-based, and neural-fuzzy myoelectric control systems. In addition, each control modality provided details on the control methods, performance, and limitations. These existing myoelectric control systems show promising outcomes, demonstrating enhanced human–robot interactions, robot intelligence, and adaptiveness to the user, task, and environment compared to traditional control systems without using electromyography. However, several challenges and limitations are commonly discussed and explored in the articles associated with myoelectric control systems applied to upper limb wearable robots. The limitations and challenges extracted from the included research articles suggest that future studies of myoelectric control systems for upper limb exoskeletons should focus on narrowing the gap between laboratory studies and clinical applications. Further studies are needed to investigate the myoelectric control system of upper limb exoskeletons in clinical environment. This chapter discusses the research questions and tasks for future research works to narrow the gap between laboratory studies and clinical applications, which include (1) the robustness of machine learning-based myoelectric control, (2) the calibration procedure of the biomechanical model-based myoelectric control system, (3) the implementation of myoelectric control systems to a high degree-of-freedom wearable exoskeleton, (4) the incorporation of safety requirements in myoelectric control systems, (5) a clinical assessment of assistive and rehabilitative wearable exoskeletons based on myoelectric control systems, and (6) the incorporation of human-centered myoelectric control. These are crucial barriers to the effective use of myoelectric controls on upper limb wearable exoskeletons, warranting further research.

### 4.1. How Can We Improve the Robustness of Machine Learning-Based Myoelectric Control Systems?

The robustness of the myoelectric control system relates to its capability to resist disturbance from and within the environment on electromyography signal [94]; such a disturbance is generally caused by muscle fatigue [95], electrode shift [96], and changes in EMG patterns over time [97]. The number of studies using machine learning (ML)-based myoelectric control systems has significantly increased over the past decade, showing promising performance from preliminary/pilot testing in laboratory settings. However, none of these ML-based myoelectric control systems have yet studied approaches to improving their robustness. To bridge the gap between experimental research and commercial/clinical applications, machine-learning-based myoelectric control systems should focus on developing an accurate control scheme under a well-controlled laboratory environment and improving the robustness under real-world environment [98].

Within the reviewed research articles that implemented machine-learning-based myoelectric control systems in this review, commonly reported issues include varying characteristics of EMG signals between different physiological conditions, noise/artifacts, and muscle fatigue that cause variants in the EMG signal, and electrode shifts during or between sessions. However, none of the research articles included in this review specifically addressed these issues. Existing studies on the myoelectric control of prosthetics, teleoperate robotic arms, and the pattern recognition of EMG signals have already investigated such issues. For example, [99] explored the property of muscle coordination in the frequency domain, and introduced a novel feature of the EMG signal to improve the accuracy in classifying movement intention from the EMG signal to incorporate variations in the EMG signal. The authors in [100] used a post-processing method to enhance the robustness of the classifier against the variant in the EMG signal caused by muscle fatigue and noise/artifacts. The authors in [101] investigated the optimal distance between EMG electrodes and the feature selection method for myoelectric pattern recognition under the impact of electrode shift, and provided solutions to improving the robustness of myoelectric pattern recognition in the presence of an electrode shift [94]. The behaviors of all time and frequency domain EMG features are evaluated to classify upper limb motions. Based on the abovementioned research, the potential approaches for improving the robustness of machine learning-based myoelectric control systems include the use of more efficient features, reducing the impact of EMG electrode shifts, and improving the data collection protocol or signal processing method to enhance the robustness of the machine learning model. Further investigation of the combination of the abovementioned robustness-improving methods and myoelectric control systems and their performance on the upper limb exoskeleton are needed. One of the open questions is to investigate the performance of an upper limb exoskeleton with a machine learning-based myoelectric control system while using different time-domain and frequency-domain features. This is because the results of our literature research indicate that most of the included research articles use the common types of features such as root mean square (RMS), mean absolute value (MAV), and zero crossing (ZC). Instead, the selection of EMG features for future studies should be expanded to all time-domain and frequency-domain features [102] and evaluate the performance of the human–exoskeleton system. With the improved myoelectric control system, the effectiveness of assisting the patient in regaining upper limb functionalities in rehabilitative training will be increased, as well as the potentiality for reducing the occurrence of musculoskeletal disorders. Furthermore, a possible shifting of EMG electrodes during use and its associated effects on myoelectric control performance should be examined. For example, while training and evaluating machine learning-based myoelectric control, the EMG electrodes can be placed slightly differently to improve the overall robustness of the model. Moreover, training protocols for a machine learning-based myoelectric control system is also less well studied For example, [103] used EMG signals collected in 21 days to train a classification model that showed that the EMG signal collected in the long term even decreased the accuracy of classification. This suggests that there is a need to investigate training protocols that generate the most optimal dataset to train machine learning-based myoelectric control systems.

In addition to the above mentioned research fields, future research should also explore the implementation of sensor fusion methods and their contributions to improving the robustness of machine learning-based myoelectric control systems. The sensor fusion of myoelectric control systems refers to the combination of sensors different from the EMG sensors to achieve a better accuracy and a better inference on the user’s movement intention. In this review, some research articles implemented sensor fusion methods to improve the accuracy of the myoelectric control system [28,30,45,52]. Some articles have benchmarked the performances of myoelectric control systems with sensor fusion by comparing them with those without sensor fusion [28]. Among the included articles that have studied sensor fusion methods, the human–robot interactive force measured by the FSR sensor, joint kinematics measured by the IMU sensor, and brain signal measured by electroencephalogram (EEG) sensor were the major signals that were combined with the EMG sensor. Identifying the optimal set of different sensor inputs and utilizing sensor fusion will lead to an enhancement of the accuracy and robustness of machine learning-based myoelectric control system. Furthermore, all such studies implemented the sensor fusion method for the machine-learning-based myoelectric control system, and the results have demonstrated the superior performance of myoelectric control systems with the sensor fusion method. However, not only the machine-learning-based myoelectric control system, but other types of myoelectric control can also work with sensor fusion methods control systems [63,104].

### 4.2. How Can We Improve the Calibration Procedure of Biomechanical Model-Based Myoelectric Control Systems?

The parameters of the human musculoskeletal models implemented in myoelectric control systems vary between users. Therefore, repetitive customization and calibration are required before each use, significantly affecting the practicality of wearable exoskeletons using biomechanical model-based control, and limiting their translation from in-lab assessment to real-world applications. The calibration of the musculoskeletal model needs additional experiments before its use as the anatomical model of the limb. For example, in [59], a motion capture system was utilized to facilitate the calibration procedure. First, the kinematic data of users were collected using the motion capture system while performing the designated motions. Then, a biomechanical simulator (OpenSim) was used to scale a generic musculoskeletal model with the captured data to calibrate the biomechanical model in the myoelectric control system. This process consumes a significant amount of time and cannot be conducted by the users themselves. Some included articles investigated different approaches to simplify the calibration procedures; for example, ref. [29] implemented a data-driven optimization method to calibrate the musculoskeletal model, and [47] measured the gravity and interaction torque under various load conditions and used them to optimize the parameters of the musculoskeletal model using the Genetic Algorithm. Both approaches have demonstrated much easier calibration procedures than model-based myoelectric control systems within the included articles [23,24,25,33,35,37,59,63,72].

Although the included articles have made many efforts to simplify the calibration procedure [29,47] and significant improvements have been achieved, implementing the generic musculoskeletal model to the myoelectric control system of the upper limb exoskeleton has not been explored. Unlike the subject-specific musculoskeletal model, which is calibrated for a single human user, the generic musculoskeletal model is calibrated using the data collected from a group of humans. Compared to the subject-specific musculoskeletal model, the generic musculoskeletal model is less accurate but has better adaptability to different human users. However, Turvey [105] studies mentioned that the performance of a generic musculoskeletal model-based myoelectric control system for a simulated upper limb prothesis was similar to the myoelectric control system with a subject-specific musculoskeletal model. The factors contributing to the reliability of generic musculoskeletal model-based myoelectric control are still unclear. However, possible factors include (1) co-adaption between human and myoelectric control systems and (2) conserved biomechanical action during motions. Therefore, instead of focusing on how to improve the calibration procedure of biomechanical model-based myoelectric control systems, future studies could also investigate the questions relevant to the generic musculoskeletal model for biomechanical model-based myoelectric control systems of upper limb exoskeletons; for example, the researchers can use different approaches to form a group of human subjects to collect the biomechanical data for a generic musculoskeletal model, and to investigate the impact of various human subject groups on the accuracy of the generic musculoskeletal model. Furthermore, future studies could also study the co-adaption between human users and biomechanical model-based myoelectric control systems. Only [9] within the selected articles studies the co-adaption between the human user and the proportional myoelectric control system, and the co-adaption between the human user and biomechanical model-based myoelectric control system is still unexplored. However, as the exoskeleton is a symbiotic robot that requires a close interaction between humans and the exoskeleton, understanding the co-adaption between human and biomechanical model-based myoelectric control systems can help researchers to improve the robustness and adaptability of myoelectric control systems, and also to accelerate the development of generic musculoskeletal model-based myoelectric control systems of upper limb exoskeletons.

### 4.3. How Can We Implement Myoelectric Control Systems for High Degree-of-Freedom (DOF) Upper Limb Exoskeletons?

Most papers have explored only a single DoF control scheme, rather than the simultaneous control of multiple DoFs, due to its relative simplicity, while a multiple DoF system could render the enhanced adaptability of the robot. Compared to a single DoF actuation control, the simultaneous control of multiple DoF is challenging in practice. Having more than a single DoF requires the exoskeleton joint motion controller to properly coordinate the desired limb motion. During complex motions, including cyclic tasks (e.g., table wiping or cutting vegetables), the frequencies of different joint movement trajectories must be synchronized to produce the robot’s desired periodic motion. However, in the conventional EMG-based control methods, e.g., where a single (or a pair of) muscle activity is mapped to a single DoF actuation, joints with only a few DoF can be controlled because of their low motion complexity. Similarly, a fuzzy controller’s rules would become convoluted with an increased number of DoF and/or periodic motions. For the more involved and higher DoF movements, such as that of the hand, a machine learning-based myoelectric muscle motion pattern recognition has proven to be a reliable method due to its characteristics previously detailed in Section 3.3.2 [54]. In addition, an EMG signal is usually not limited to the data generated by one muscle, and could also be reflecting the activity of the neighboring muscles in the area, the so-called crosstalk issue [106]. Therefore, the fact that different muscles are associated with different motions leads to difficulty in the unequivocal association of the EMG signal with motion. Estimating a single joint angle with little influence of a change in other joints’ positions and reducing crosstalk is the best practice [24]. Additionally, high computational costs remain another barrier in the physical implementation of such detection methods into real-time embedded systems.

To provide effective assistance in ADLs, an upper limb wearable exoskeleton must have a high number of DoFs, and a controller to coordinate those motions while requiring the user to put in a minimum of effort. Therefore, dimensionally reducing the control problem can be a possible approach. In one study, to dimensionally reduce the essential control inputs, a 10-DoF (independently actuated) whole-hand exoskeleton took advantage of kinematic synergies—a collection of relatively independent degrees of freedom behaving as one functional unit [105]—enabling the researchers to test different synergies or control approaches [56]. They experimented with three synergies related to the thumb, index, and remaining three fingers’ independent flexions/extensions. Researchers have successfully controlled the hand exoskeletons to perform common hand gestures for all the fingers, excluding the thumb, as the complex motion of the thumb imposes difficulties on the mechanical structure design [44,65]. It remains a challenge to design mechanisms for all fingers to build a complete hand exoskeleton that provides rehabilitation for different grasping movements.

Finally, the number of actuators increases with the number of DoFs, causing limitations in wearability and portability as it increases the weight, cost, and size. Therefore, reducing the number of DoFs; for example, mechanically coupling a few joints with small ranges of motions or requiring low actuation forces, can make the device simpler and more lightweight. One drawback of this solution is that it can limit the flexibility and possible motion patterns of the system. Another possible solution can be having fewer actuators than the number of DoFs, which is known as the under actuation strategy [44].

### 4.4. How to Incorporate Safety Requirements in Myoelectric Control Systems?

Safety is a critical requirement for upper limb exoskeletons, particularly for active powered systems. For assistive and rehabilitative applications, it is essential to ensure the safety of the patients so that these robots will not cause a risk of injury during use. Existing exoskeletons in the literature mainly focus on implementing safety measures in mechanical design, such as installing mechanical stops, rotation limits, or force limits to guarantee that there is no excessive range of motion movements or excessive force applied to the user [2].

However, such mechanisms cannot always fully warrant the safety of the user in the presence of unknown large parameter variances, hardware failures, or actuator defects [107]. Therefore, control strategies that can effectively compensate for different uncertainties and external load disturbances might substantially contribute towards improving the safety of the user while performing motions/tasks wearing the robotic exoskeleton. Given the inherent variability of EMG signals (arising from changes in arm posture, electrode repositioning, fatigue etc.), one possible approach can involve applying data fusion techniques on EMG signals to reduce the potential errors of motion estimation. In [108], two data fusing algorithms, Variance Weighted Average (VWA) and Decentralized Kalman Filter (DKF), were proposed for the myoelectric control of a robotic arm, in order to improve its reliability under electrode faults and noisy environments.

For machine learning- or deep learning-based myoelectric control systems, implementing post-processing techniques, such as multiwindow smoothing and confidence estimation, may also improve the reliability of the controller [109]. Multiwindow smoothing approaches can reduce potential estimation errors by utilizing EMG signals from successive sliding windows [110], while confidence estimation is used to analyze the confidence of classification results so that uncertain decisions can be detected and eliminated [111]. Furthermore, using adaptive control with real-time learning and prediction can also help in increasing the fault tolerance of the proposed system [112]. Future studies may consider focusing on how to improve the safety measures in wearable exoskeletons from both mechanical and controller design perspectives to enhance the reliability and fault-tolerance of upper limb exoskeletons for real-world applications.

### 4.5. How Can We Improve Experimental Validation and Clinical Assessment Methods for Assistive and Rehabilitative Exoskeletons Based on Myoelectric Control Systems?

The myoelectric control systems reviewed in the literature were tested in laboratory settings, mostly on able-bodied users. Although many studies showed good preliminary results with healthy subjects, to transition from laboratory to real-world assistive and rehabilitative applications, these prototypes need to be further tested on large-scale clinical populations to address inter- and intra-subject variability issues. Because the voluntary muscle contraction level and muscle fatigue endurance may vary between healthy and neurologically impaired subjects; thus, it might impact on the efficacy of the proposed control method in assisting the intended motion/task of subjects with motor disabilities with high accuracy and reliability, despite achieving desired results with healthy users. For example, in [36], the overall average controller accuracy for a hand exoskeleton was 98.1±4.9% among neurologically intact subjects. However, when tested on SCI subjects, the controller accuracy was found to be lower (90.0±13.9%). Similarly, in [64], the average classification accuracy of the proposed EMG-based classifier for an upper limb exoskeleton was lower among the SCI group (85%-95% for single-DoF and 60% for multi-DoF) compared to able-bodied participants (99% for single-DoF and 90% for multi-DoF). These findings highlight the importance of experimental validation of myoelectric control systems on a clinical population to evaluate the controller performance in the presence of neurological impairment. Furthermore, while interpreting study outcomes, the different level of motor impairment among clinical subjects should also be taken into account (as performed in [32]), particularly in those cases where patients with different neurological conditions or motor function levels are recruited for testing.

Experimental validation methods for rehabilitative exoskeletons in the reviewed literature have mostly been limited to evaluating trajectory tracking performance, movement execution velocity, user’s effort, and joint torque estimation for one session of lab assessment. Although these outcome measures may indicate the functionality of the physical model and the efficacy of the control system in assisting the user in performing a certain movement or task, it might not completely reflect the effectiveness of the robot-assisted training in improving the motor function of subjects with neuro-muscular disorders. Since clinical validation studies of myoelectric control systems for upper limb exoskeletons are still in their infancy, the focus should be given to performing experimental validation with control groups at least, to provide stronger evidence for the effectiveness of robot-assisted training. In addition, there is a possibility that the classification accuracy of ML-based myoelectric control might not be strongly associated with the training effectiveness. For example, in [113], the real-time testing of myoelectric control prosthetics on trans-radial amputees with virtual arm showed that the completion rate of predefined hand motions was 55% on average, despite obtaining 85% classification accuracy for the pattern recognition method. This suggests that this proposed pattern recognition-based myoelectric control was not able to reliably control real-time hand grasp, even with good classification accuracy.

Clinical assessment tests are useful in evaluating the efficacy of myoelectric control-based exoskeletons in achieving desired outcomes among neurologically impaired patients. For example, in [41], the Sollerman hand function test was used to assess the performance of a hand exoskeleton in improving hand functions required for common ADLs among SCI subjects. Also, in [15], the Fugl-Meyer Assessment (FMA) and the Action Research Arm Test (ARAT) were used to evaluate the effect of an upper limb exoskeleton on upper limb functions and hand functions among chronic stroke patients, respectively. The results showed that the motor functions of the subjects on the hand and upper limb significantly improved after 20 sessions of robot-assisted hand functions task training. Therefore, future studies should consider incorporating multiple robot-assisted training sessions, followed by clinical assessment tests, to evaluate the robustness and effectiveness of rehabilitative upper limb exoskeletons in restoring upper limb functions in patients with motor disabilities.

### 4.6. How Can We Develop a Human-Centered Myoelectric Control System Instead of a Task-Centered Myoelectric Control System?

The research literature included in this systematic review has shown innovative ideas for using EMG signals to control an upper limb wearable robot. However, the presented control modalities can be broadly categorized as using a machine learning model or a musculoskeletal model, using EMG for the continuous control of assistive torque (proportional), and starting the assistance of a wearable robot if the EMG signal satisfies certain conditions (neural fuzzy and threshold). Despite these articles demonstrating promising results, these myoelectric control systems are developed to map the user’s movement intention from the EMG signal to a specific task, and then to control the wearable robot to complete this task. The wearable robots controlled by such types of myoelectric control systems only focus on finishing the assigned tasks, and are unable to perceive the feedback of the user. However, the wearable robot and human physically interact in a bidirectional approach; therefore, understanding the feedback of the human by developing a human-based myoelectric control system can improve adaption and synchrony between the human and the wearable robot.

Different from the existing literature, the human-based myoelectric control system should focus on fulfilling the human’s motion intention, which can be achieved by minimizing human effort (e.g., metabolic cost, muscle activation/fatigue, etc.). For example, when controlling an upper limb wearable robot to help the human to lift a heavy load, the myoelectric control system should no longer estimate the load from the EMG signal and control the robot to output the corresponding assistive force to compensate for the gravitational load. Instead, the myoelectric control system will control the wearable robot to output a proper amount of assistive force to minimize the amplitude of the human’s EMG signal. Compared to the conventional control approach, the human-based myoelectric control system is adaptive to the variant of EMG signal, which reduces the dependency on calibration. As a matter of fact, such an approach has been introduced by [114] to control an upper limb wearable robot to compensate for the gravity of the human arm. However, ref. [114] only validates the proposed control system in a simulation environment. Within the included literatures, ref. [40] used a model-based reinforcement learning algorithm to learn the control strategy of the upper limb wearable by minimizing the amplitude of EMG signal. However, such a model-based reinforcement learning algorithm requires a significant amount of computational power. Furthermore, ref. [35] studied a myoelectric control system based on a similar concept, but used the musculoskeletal model to convert the EMG signal to the muscle force before using it as a control command. Moreover, the abovementioned myoelectric control systems are only developed for human power augmentation by compensating gravitational loads. Therefore, the applications of human-based myoelectric control are largely unexplored. Further studies should consider exploring the application of such control schemes on more dexterous motions, more complicated scenarios such as rehabilitative training, and the control system that requires less computational power.

## 5. Conclusions

The myoelectric control systems have shown their efficacy for use in the upper limb exoskeletons to enhance human–robot dynamics and interactions. This review focused on identifying and categorizing key control strategies employed in myoelectric control systems implemented on upper limb exoskeletons. The overview of each myoelectric control system was presented, highlighting their control performance and accuracy, as well as their limitations and challenges. Some future research directions are proposed: improving adaptiveness across different users, tasks, and environments, myoelectric control systems for multi-joints and/or bilateral arm assistance, improving robustness against the disturbance in the real-world application, developing a human-centered myoelectric control system, and improving its safety features through a human subject evaluation process. It was noted that comparably less work has been performed to exploit a human-centered myoelectric control system, and fewer validated models exist in the literature. This warrants further research to assess the applicability of a human-centered myoelectric control system to control upper limb wearable exoskeletons. Further, all myoelectric control systems discussed in this review were evaluated in laboratory settings, and mostly on healthy able-bodied individuals, limiting their implications on clinical applications. Nevertheless, the rapid evolution and the many variations of myoelectric control systems observed in recent years suggest that it is the most promising strategy for the intelligent, adaptive, robust, and human-in-loop control of upper limb exoskeletons.

## Figures and Tables

**Table 1:** Description of search query used in each database.

Database	Search Query
PubMed	(((myoelectric OR electromyography OR EMG) AND (upper limb OR elbow OR shoul-der OR hand OR finger OR wrist)) AND (exosuit OR exoskeleton)) AND ((“2011/01/01” [Date—Publication]: “2021/12/31” [Date—Publication]))
Web of Science	(((TS = (upper limb OR upper body OR wrist OR elbow OR shoulder OR finger OR hand)) AND (TS = (electromyography OR emg OR semg OR surface electromyography OR myoelectric)) AND (TS = (exoskeleton OR exosuit)) AND PY = (2011–2021)) NOT TS = (Passive)) NOT TS = (Lower Limb OR Hip OR Knee)
IEEE Xplore	(“All Metadata”: upper limb OR “All Metadata”: upper body OR “All Metadata”: elbow OR “All Metadata”: shoulder OR “All Metadata”: wrist OR “All Metadata”: hand OR “All Metadata”: finger) AND (“All Metadata”: electromyography OR “All Metadata”: emg OR “All Metadata”: EMG OR “All Metadata”: myoelectric) AND (“All Metadata”: exosuit OR “All Metadata”: exoskeleton)

**Table 2:** General summary of the research papers included in this review. Corresponding to Citation of document, Author Info., Publish Year, Included Body Segments, Type of Myoelectric Control System, Portability, DoF, Application of Exoskeleton, and Characteristics of Experimental Subject.

No.	Ref and Author	Year	Body Segment	Control Method	Device Portability	DoF	Application of Device	Experimental Subject
1	Ho et al. [15]	2011	Hand	Threshold	Portable	10	Rehabilitation	8 chronic stroke subjects
2	Lenzi et al. [9]	2012	Elbow	Proportional	Fixed	1	Augmentation and Assistance	10 healthy subjects
3	Gopura and Kiguchi [16]	2012	Shoulder, Elbow, Wrist	Neural Fuzzy	Fixed	6	Rehabilitation	1 healthy subject
4	Kiguchi and Hayashi [17]	2012	Shoulder, Elbow, Wrist	Neural Fuzzy	Fixed	7	Rehabilitation and Assistance	3 healthy subjects
5	Pang et al. [18]	2012	Hand	Machine Learning	Portable	1	Rehabilitation	3 healthy subjects
6	Delph et al. [19]	2013	Hand	Proportional	Portable	1	Rehabilitation	Not Specified
7	Loconsole et al. [20]	2013	Hand	Machine Learning	Portable	1	Rehabilitation	1 healthy subject
8	Su et al. [21]	2013	Elbow	Machine Learning	Fixed	3	Rehabilitation	1 healthy subject
9	Ngeo et al. [22]	2013	Hand	Machine Learning	Portable	3	Assistance	1 healthy subject
10	Ramos and Meggiolaro [23]	2014	Elbow	Model Base	Portable	2	Augmentation	1 healthy subject
11	Kawase et al. [24]	2014	Elbow, Wrist	Model Base	Portable	6	Rehabilitation	6 healthy subjects, 1 SCI patient
12	Loconsole et al. [25]	2014	Elbow	Model Base	Fixed	1	Rehabilitation	1 healthy subject
13	Li et al. [26]	2014	Elbow, Wrist	Machine Learning	Fixed	2	Assistance	5 healthy subjects
14	Li et al. [ 27]	2014	Elbow	Machine Learning	Portable	1	Rehabilitation	6 healthy subjects
15	Kirchner et al. [28]	2014	Elbow, Wrist	Machine Learning	Portable	4	Rehabilitation	8 healthy subjects
16	Buongiorno et al. [29]	2015	Shoulder, Elbow	Model Base	Fixed	4	Rehabilitation	3 healthy subjects
17	Riener and Novak [30]	2015	Elbow	Threshold	Fixed	7	Rehabilitation	3 healthy subjects
18	Krasin et al. [31]	2015	Elbow	Threshold	Portable	1	Augmentation	Not specified
19	Leonardis et al. [32]	2015	Hand	Machine Learning	Portable	1	Rehabilitation	6 healthy subjects, 2 chronic stroke patients
20	Ullauri et al. [33]	2015	Elbow	Model Base	Fixed	1	Rehabilitation	2 healthy subjects
21	Triwiyanto et al. [34]	2016	Elbow	Proportional	Fixed	1	Rehabilitation	Not specified
22	Peternel et al. [35]	2016	Elbow	Model Base	Fixed	1	Rehabilitation and Assistance	8 healthy subjects
23	Accogli et al. [8]	2017	Wrist, Elbow	Machine Learning	Fixed	4	Assistance	1 healthy subject
24	Lu et al. [36]	2017	Hand	Machine Learning	Fixed	5	Rehabilitation	8 healthy subjects; 2 SCI subjects
25	Li et al. [37]	2017	Elbow	Model Base	Fixed	2	Assistance	1 healthy subject
26	Hosseini et al. [38]	2017	Elbow	Threshold	Portable	2	Assistance	1 healthy subject
27	Mghames et al. [39]	2017	Elbow	Proportional	Portable	1	Augmentation	1 healthy subject
28	Hamaya et al. [40]	2017	Elbow	Machine Learning	Fixed	1	Assistance	5 healthy subjects
29	Yun et al. [41]	2017	Hand	Machine Learning	Portable	8	Augmentation	2 SCI Patients
30	Irastorza-Landa et al. [42]	2017	Wrist	Machine Learning	Portable	7	Rehabilitation	8 healthy subjects
31	Lambelet et al. [43]	2017	Wrist	Proportional	Portable	1	Rehabilitation	1 healthy subject
32	Lince et al. [44]	2017	Hand	Proportional	Portable	4	Rehabilitation	8 healthy subjects
33	Copaci et al. [45]	2018	Elbow	Threshold	Portable	1	Rehabilitation	1 healthy subject
34	Zeng et al. [46]	2018	Hand	Machine Learning	Portable	6	Rehabilitation	25 healthy subjects
35	Buongiorno et al. [47]	2018	Elbow	Model Base	Fixed	4	Augmentation and Assistance	7 healthy subjects
36	Cisnal et al. [48]	2019	Hand	Proportional	Portable	10	Rehabilitation	Not Specified
37	Jana et al. [49]	2019	Hand	Machine Learning	Portable	2	Rehabilitation	1 healthy subject
38	Trigili et al. [50]	2019	Elbow, Shoulder	Machine Learning	Fixed	4	Augmentation	10 healthy subjects
39	Lei [51]	2019	Elbow, Wrist	Machine Learning	Portable	1	Rehabilitation	4 healthy subjects
40	Wu et al. [52]	2019	Elbow	Machine Learning	Portable	1	Rehabilitation and Assistance	5 healthy subjects
41	Xiao [53]	2019	Elbow	Machine Learning	Fixed	1	Rehabilitation	9 healthy subjects
42	Lu et al. [54]	2019	Hand	Machine Learning	Portable	5	Rehabilitation	12 SCI Patients
43	Secciani et al. [55]	2019	Hand	Machine Learning	Portable	4	Assistance	1 patient with hand impairment
44	Burns et al. [56]	2019	Hand	Machine Learning	Portable	10	Rehabilitation	5 healthy subjects
45	Lu et al. [57]	2019	Elbow	Machine Learning	Portable	1	Assistance	5 healthy subjects
46	Li et al. [58]	2020	Elbow	Machine Learning	Portable	1	Assistance	10 healthy subjects
47	Lotti et al. [59]	2020	Elbow	Model Base	Portable	1	Assistance	6 healthy subjects
48	Hosseini et al. [60]	2020	Elbow	Threshold	Portable	2	Assistance	4 healthy subjects
49	Da Silva et al. [61]	2020	Elbow	Proportional	Portable	2	Assistance	1 healthy subjects
50	Treussart et al. [62]	2020	Elbow	Proportional	Fixed	1	Augmentation	10 healthy subjects
51	Liu et al. [63]	2020	Elbow	Model Base	Fixed	1	Rehabilitation and Assistance	5 healthy subjects
52	McDonald et al. [64]	2020	Elbow	Machine Learning	Portable	4	Rehabilitation	10 healthy subjects, 1 SCI patient
53	Castiblanco et al. [65]	2021	Hand	Neural Fuzzy	Portable	4	Rehabilitation	4 stroke patients and 3 healthy subjects
54	Liu et al. [66]	2021	Elbow	Machine Learning	Portable	1	Rehabilitation	20 healthy subjects
55	Yang et al. [67]	2021	Elbow	Machine Learning	Portable	1	Rehabilitation	10 healthy subjects
56	Zhou et al. [68]	2021	Shoulder	Machine Learning	Fixed	5	Rehabilitation	18 healthy subjects
57	Cisnal et al. [69]	2021	Hand	Threshold	Portable	5	Rehabilitation	10 healthy subjects
58	Xiao et al. [70]	2021	Hand	Machine Learning	Fixed	7	Rehabilitation	9 healthy subjects
59	Liu et al. [71]	2021	Shoulder, Elbow	Proportional	Portable	2	Augmentation	3 healthy subjects
60	Xie et al. [72]	2021	Hand	Model Base	Portable	8	Rehabilitation	Not specified

## Data Availability

Not applicable.
